# Gene Signatures and Prognostic Values of N6-Methyladenosine Related Genes in Ovarian Cancer

**DOI:** 10.3389/fgene.2021.542457

**Published:** 2021-08-18

**Authors:** Zhijing Na, Ling Fan, Xiuxia Wang

**Affiliations:** ^1^Center of Reproductive Medicine, Shengjing Hospital of China Medical University, Shenyang, China; ^2^Nursing Department, Shengjing Hospital of China Medical University, Shenyang, China

**Keywords:** subgroup, prognosis, m^6^A-related genes, m^6^A RNA methylation regulators, ovarian cancer

## Abstract

N6-Methyladenosine (m^6^A) is one of the most prominent modification regulating RNA processing and metabolism. Increasing studies have illuminated the vital role of m^6^A methylation in carcinogenesis. However, little is known about the interaction between m^6^A-related genes and survival of ovarian cancer (OC) patients. The purpose of this study was to obtain more reliable m^6^A-related genes that could be used as prognostic markers of OC using bioinformatics analysis performed on the RNA-seq data of OC. Gene expression datasets of all m^6^A-related genes as well as corresponding clinical data were obtained from the International Cancer Genome Consortium (ICGC) and The Cancer Genome Atlas (TCGA) databases. We detected differential expressed m^6^A-related candidate genes as well as their relationship and interaction. m^6^A RNA methylation regulator ALKBH5 and 35 m^6^A-related genes are dysregulated in OC. A gene set that could be used as a potential independent prognostic risk feature was further screened including NEBL, PDGFRA, WDR91, and ZBTB4. The results of mRNA expression analysis by PCR were consistent with those of bioinformatics analysis. We applied consensus clustering analysis on the expression of the four prognostic genes and obtained four OC subgroups TM1-TM4. There were significant differences in age, stage and grade among the subgroups, and the overall survival (OS) as well as Disease-free survival (DFS) of TM2 group were shorter than those of the other three groups. Further GO and KEGG enrichment analysis indicated that these differential genes were closely related to biological processes and key signaling pathways involved in OC. In summary, our study has indicated that m^6^A-related genes are key factors in the progression of OC and have potential effects on the prognostic stratification of OC and the development of treatment strategies.

## Introduction

Ovarian cancer (OC) ranks the seventh most common cancer worldwide, with a total incidence of 239,000 each year (Bray et al., 2018). It is the leading cause of gynecologic cancer-related deaths among women, causing 152,000 deaths yearly (Torre et al., 2018). Due to the missing early symptoms and the absence of effective early detection strategies, approximately 70% of OC patients were diagnosis at advanced stage presenting with metastases (Oldak et al., 2019). Although the treatment for OC has been greatly improved in recent decades, the recurrence is frequent and the 5-year overall survival rate remains poor (Coleman et al., 2019). Hence, it’s urgent to explore specific early diagnostic and prognostic biomarkers and improve the unfavorable prognosis of patients suffering from OC.

N6-Methyladenosine (m^6^A) modification, the most prominent chemical modifications in eukaryotic mRNA and noncoding RNA (lncRNA), is a dynamic reversible process that regulates RNA processing and metabolism (Deng et al., 2018; Yu J. et al., 2018). m^6^A modification on RNAs was labeled by methyltransferases (writers), preferentially recognized and transmitted by binding proteins (readers), and erased by demethylases (erasers) (Zhao et al., 2020; Jiang et al., 2021). METTL3, METTL14, WTAP, KIAA1429, ZC3H13, and RBM15 are writers that catalyze the methylation of m^6^A into RNAs (Wang T. Y. et al., 2020). Readers are capable of selectively recognizing m^6^A-modified RNAs to mediate the translation and degradation of RNAs, including HNRNPC, YTHDC1, YTHDC2, YTHDF1, and YTHDF2 (Shi et al., 2019). Finally, FTO and ALKBH5, considered as erasers, can remove the methyl group from target RNAs to achieve the dynamics and reversibility of the m^6^A modification process (Livneh et al., 2020).

Currently, increasing studies have demonstrated the roles of m^6^A methylation in various carcinogenesis processes including cell self-renewal (Dai et al., 2018), differentiation (Sun et al., 2019), cell proliferation (Liu et al., 2020), migration (Cheng et al., 2019; Liu et al., 2020), invasion (Cheng et al., 2019), autophagy (Wang et al., 2019), apoptosis (Ianniello and Fatica, 2018), and metastasis (Yue et al., 2019). For instance, overexpression of METTL3 in AML significantly inhibits cancer cell differentiation and apoptosis via activating PI3K/AKT pathway (Ianniello and Fatica, 2018). ALKBH5 erases m^6^A modification of tumor suppressor gene FOXM1 to promote cancer cell maturation and tumorigenicity in glioblastoma (Zhang et al., 2017). The aberrant m^6^A modifications were reported to contribute to multiple cancers, including lung cancer (Li et al., 2019), breast cancer (Niu et al., 2019), glioblastoma (Zhang et al., 2017), acute myeloid leukemia (AML) (Ianniello and Fatica, 2018), liver cancer (Cheng et al., 2019), gastrointestinal cancer (Ni et al., 2019; Sun et al., 2019), endometrial cancer (Liu et al., 2018), et al. Thus, m^6^A has great potential as promising markers in the diagnosis, prognosis and personalized targeted therapies of cancers. Since the effects of m^6^A-related genes in OC have not been mentioned yet, we conducted our study to shed light on the expression pattern, prognostic value and potential mechanisms of m^6^A-related genes in OC patients.

## Materials and Methods

### The Expression Pattern of m^6^A-Related Genes in OC Patients

#### Data Source

We downloaded the RNA-seq transcriptome data and corresponding clinical information of 308 OC samples from the TCGA database from the UCSC’s xena database^1^. At the same time, RNA-seq data from 200 OC samples from the ICGC database independent of the samples used in the TCGA dataset were also obtained. The original RNA-seq data was standardized data, and it was uploaded online (DOI: 10.6084/m9.figshare.14399900), and the all TCGA codes of the patients used in this study was displayed in Supplementary Table 1. The corresponding clinical information of the validated ovarian cancer samples were downloaded, as shown in Table 1.

**TABLE 1:** The clinical information of the validated ovarian cancer samples.

**Datasets**	**data_source**	**Parameter**	**Subtype**	**Patients n**
**Clinical pathological characteristics of patients with OC**				
TCGA	UCSC_xena	Age (years)	>58	147
			≤58	161
		Gender	Female	308
		Pathologic stage	I	1
			II	22
			III	245
			IV	38
			Unknown	2
		Grade	G1	1
			G2	37
			G3	261
			G4	1
			Unknown	8
		new_neoplasm_event_type	Metastasis	1
			Locoregional Disease	4
			Recurrence	146
			Progression of Disease	12
			Unknown	145
ICGC	UCSC_xena	Age (years)	>59	97
			≤59	103
		Gender	Female	200
		disease_status_last_followup	complete remission	49
			Progression	42
			Unknown	109

#### Identification of Functionally m^6^A-Modified Genes

We first collected some m^6^A RNA methylation related genes from the known literature, which could be divided into three types according to the role they played in the methylation process: methyltransferases, binding proteins (readers) and erasers. A total of 21 methylation related genes were obtained, they are: METTL3, WTAP, ZC3H13, RBM15, METTL14, YTHDC1, YTHDC2, YTHDF2, YTHDF3, HNRNPA2B1, HNRNPC, HNRNPG, FTO, ALKBH5, IGF2BP2, IGF2, IGF2, ZNF217, elF3H, elF3J (Yang et al., 2018; Lence et al., 2019). Then, in the existing m^6^A database m^6^Avar^2^, there were 296 m^6^A-related genes related to OC disease. The two data sets of m^6^A-related genes were integrated together, after removing duplicates and genes that had no expression value in the sample or expression values that were less than 80% of all samples. The resulting m^6^A-related gene set contained a total of 267 m^6^A-related genes, including 18 m^6^A RNA methylation regulators and 249 m^6^A-related genes.

#### Expression Data

**FIGURE 1:**
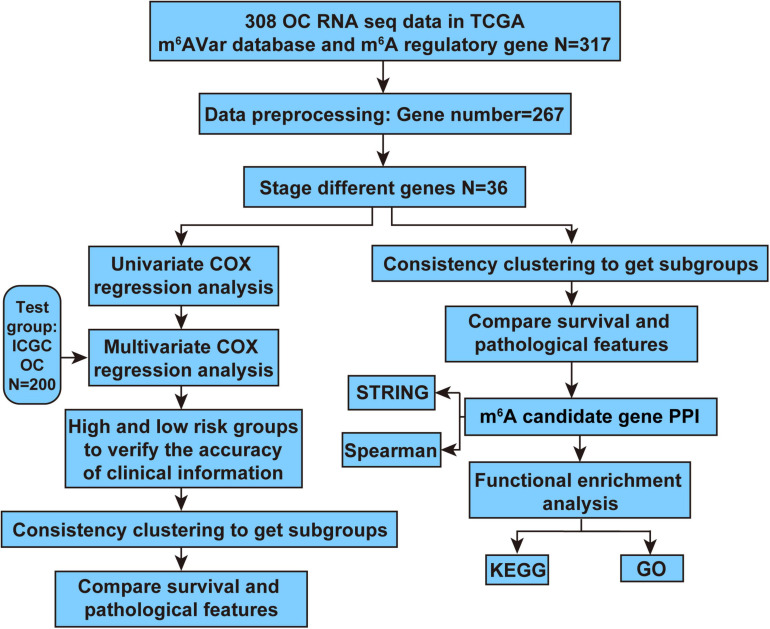
Analysis workflow of this study.

In order to determine the m^6^A RNA methylation regulatory factors that were differentially expressed according to different stages of OC, we compared the expression values of the TCGA data using the one-way ANOVA (*p* ≤ 0.05) with R language, version 3.6.0. UQ-FPKM (Upper-Quartile Fragments Per Kilobase Million reads) normalization allows for cross-sample comparison, thus we conducted a one-way ANOVA of the previously normalized absolute expression values in this study. The analysis workflow of our study is shown in Figure 1. First, we preprocessed the TCGA data and selected pathological features of stage (Stage I, Stage II, Stage III, Stage IV) from RNA-seq expression data set of m^6^A RNA methylation regulators and m^6^A-related genes related to OC, a data matrix containing 267 genes and 306 samples was obtained.

### The Prognostic Value of m^6^A-Related Genes in OC Patients

#### Consensus Clustering

Consensus clustering analysis was conducted to classify OC samples, utilizing the m^6^A-related candidate gene set related to the prognosis of OC. Patients were then divided into subgroups according to age, stage, grade, new neoplasm event type, BRCA1 mutation status, BRCA2 mutation status, 19q13.2 CNV mutation and 19q13.42 mutation. Then, we conducted comparative analysis of subgroup survival differences.

#### Survival Analysis

Univariate Cox regression analysis was performed for each functional m^6^A modifier using R “Survival” package to screen gene sets related to prognosis, and genes with *p* ≤ 0.05 were candidate genes with potential independent prognostic efficacy. We applied LASSO regression to further screen for potential prognostic risk characteristics. Multivariate Cox regression analysis of risk characteristics was conducted to further screen for genes that were significantly related to survival. Meanwhile, risk scores were calculated based on the constructed risk characteristics (Supplementary Table 2). Each OC Risk score is calculated as follows: Risk Score = $\sum_{i=1}^{n}{\text{Coef}}_{i}*{\text{Exp}}_{i}$ (Coef: regression coefficient; Exp: expression value; n: total number of samples; i:the identifier of the ith selected sample). Next, we split the patients with OC into low- and high-risk groups in terms of median risk score, and modeled these two categories as continuous variables to obtain the Hazard Ratio. Then, Kaplan-Meier test was adopted to test the significance of survival curves, and survival curves were drawn. Similarly, we analyzed the survival of OC samples in different clusters and compared the differences in survival between different subgroups. The predictive power of risk characteristics for 1-5-years survival estimates was achieved using nomogram. Nomograms utilize biological and clinical variables (such as tumor grade and patient age) to graphically depict statistical prognostic models that generate the likelihood of clinical events (such as cancer recurrence or death) for a given individual (Balachandran et al., 2015). The nomogram can visually display the results of Cox regression analysis.

### The Potential Mechanisms of m^6^A-Related Genes in OC Patients

#### Construction of Interaction Networks

The interactions between the m^6^A-related genes were analyzed and proved with STRING database^3^. Meanwhile, correlations with a correlation coefficient threshold |r| > = 0.3 and a rank-sum test *p* ≤ 0.01 were selected using Spearman analysis.

### Functional and Pathway Enrichment Analysis

To analyze potential functions and pathways of m^6^A RNA methylation related genes in OC, Enrichr^4^ was used for Gene Ontology (GO) and Kyoto Encyclopedia of Genes and Genomes (KEGG) pathway enrichment analysis of m^6^A-related genes related to OC.

### Validation of Clinical Samples

#### Tissue Samples

For tissue microarray (TMA) cohort, tumor tissues including 8 OC tissue specimens and 8 normal ovarian tissue specimens, were obtained from May 2020 to July 2020 at the Shengjing Affiliated Hospital of China Medical University, China. None of the patients was administered any chemotherapy, immunotherapy, or radiotherapy prior to surgery. Patients with other types of malignant tumors, cardiovascular and cerebrovascular diseases, and mental illness were also excluded. Our study was approved by the Health Research Ethics Board of the Shengjing Affiliated Hospital of China Medical University, and all the cases were pathologically confirmed. The volume of a single tissue sample was about 0.5 cm^3^. Tissue samples were sharp dissected during surgery and quickly frozen with liquid nitrogen within 15 min after restriction, and then stored at −80∘C until RNA extraction.

#### Real-Time PCR Array

Trizol reagent (Invitrogen, CA, United States) was utilized to extract total RNA according to the manufacturer’s protocol. For the quantification of 4 m^6^A prognostic risk model genes, total RNA was then reverse-transcribed into the complementary DNAs (cDNAs) with the PrimeScript^TM^ RT Reagent Kit with gDNA Eraser (TaKaRa, Dalian, China) and amplified by GoTaq^®^ qPCR Master Mix (Promega, Madison, WI, United States) using the ABI ViiA 7 Real-time PCR system (Applied Biosystems, United States). The specific primer sequences are listed in Supplementary Table 3. GAPDH was used as an internal control for the normalization of Gene expression. 2^–ΔΔCT^ method was used to calculate the relative fold change of expression for samples.

### Statistical Analysis

#### Statistical Analysis

We performed statistical analyses using SPSS 23.0 software (IBM Corp., Armonk, NY, United States) and GraphPad Prism 7 (San Diego, CA, United States). When the *p*-value (two-sided) ≤ 0.05, the difference was considered statistically significant.

## Results

### Expression Analysis of m^6^A RNA Methylation Regulators and m^6^A-Related Genes

Through univariate analysis of m^6^A-related genes related to OC in samples with different stages, a total of 36 candidate genes with significant differences in expression between different stages were selected from 267 m^6^A-related genes related to OC (*p* ≤ 0.05), of which 1 was an m^6^A methylation regulator ALKBH5 (Supplementary Figure 1).

### Risk Model and Prognostic Analysis of m^6^A-Related Genes

**FIGURE 2:**
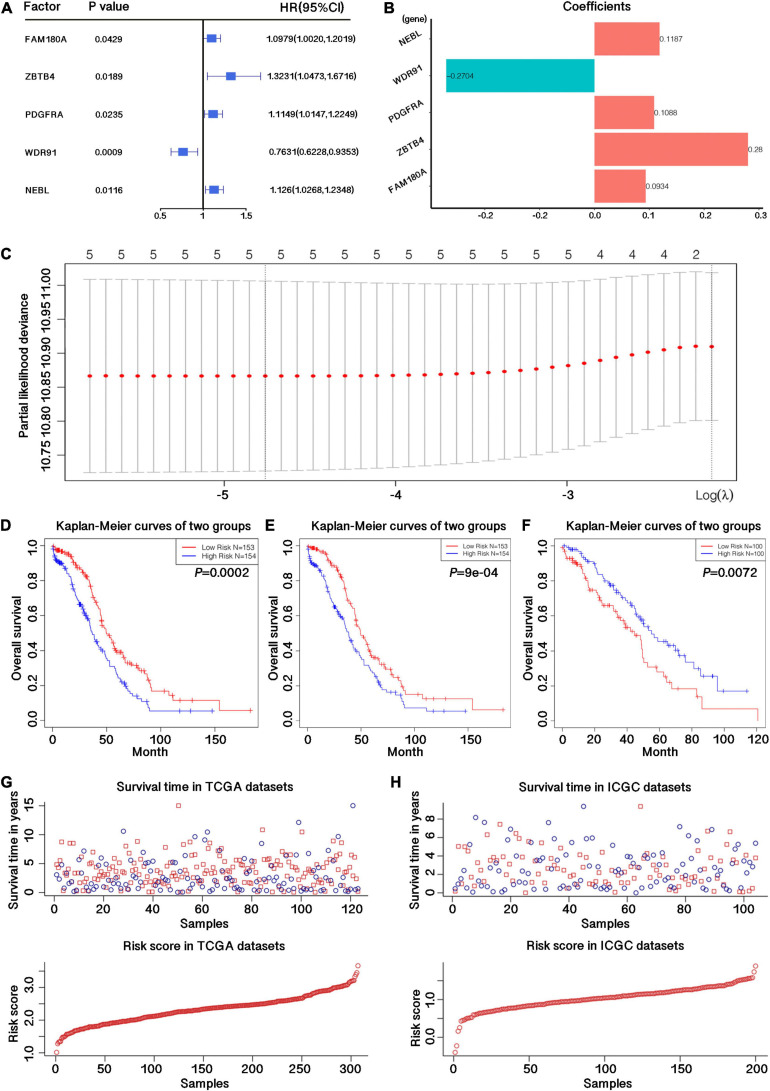
Construction of the prognostic signature with four m^6^A-related genes. **(A)** *p*-value, Hazard Ratio value and confidence interval for all risk genes with potential independent prognostic efficacy obtained by univariate Cox regression analysis. **(B)** Regression coefficients of 5 m^6^A related genes obtained by univariate Cox regression analysis. **(C)** LASSO regression map of 5 m^6^A related genes. **(D)** Survival curve drawn by dividing the TCGA training datasets into high and low risk groups based on the risk score calculated by the feature matrix constructed from the 5 candidate m^6^A-related genes. **(E)** Survival curve drawn by dividing the TCGA training datasets into high and low risk groups based on the risk score calculated by the feature matrix constructed from the 4 candidate m^6^A-related genes. **(F)** Survival curve drawn by dividing the ICGC validation datasets into high and low risk groups based on the risk score calculated by the feature matrix constructed from the 4 candidate m^6^A-related genes. **(G)** Survival time and risk store in TCGA training datasets. **(H)** Survival time and risk store in ICGC validation datasets.

In order to study the prognostic role of genes related to m^6^A RNA methylation regulation in OC, we used 306 samples with survival information in the TCGA dataset as the training data set, and all 36 related candidate genes were preliminarily screened for prognostic risk characteristics using Cox univariate regression analysis. We have initially obtained 5 genes that had potential effects on the survival of the samples. The expression level of WDR91 was positively correlated with the survival of OC patients, while the expression levels of NEBL, PDGFRA, ZBTB4, and FAM190A were negatively correlated with the survival of OC patients. The corresponding *p*-value and Hazard Ratio values of these 5 genes are shown in Supplementary Table 4 and Figure 2A, and the regression coefficients are shown in Figure 2B. LASSO regression analysis further proved that these five genes could constitute risk characteristics and the regression coefficients were obtained (Figure 2C). According to the median risk score calculated, samples were segregated into low- and high-risk groups, and survival differences between these two groups were statistically significant (*p* = 0.0002; Figure 2D). In order to eliminate the interference of other factors, we performed multivariate Cox regression analysis of candidate risk characteristics on these 5 genes using clinicopathological features such as age, stage, grade, etc. And 4 genes with significant impact on sample survival were obtained (*p* ≤ 0.05), respectively, NEBL, PDGFRA, WDR91, and ZBTB4. And these 4 genes could be used as independent prognostic markers. Subsequently, samples were split into low- and high-risk groups, and meaningful differences in survival were observed between the two groups (*p* = 0.0009; Figure 2E).

Moreover, we used 200 samples from the ICGC test dataset to further validate the stability of the risk model and the potential independent prognostic efficacy of m^6^A-related genes. The results demonstrated that these genes could effectively distinguish the survival of low- and high-risk groups (*p* = 0.0072; Figure 2F). The sample survival and the model risk score in the TCGA training set and the ICGC validation set are shown in Figures 2G,H.

### Validation of TCGA Expression Results Using Clinical Specimens

**FIGURE 3:**
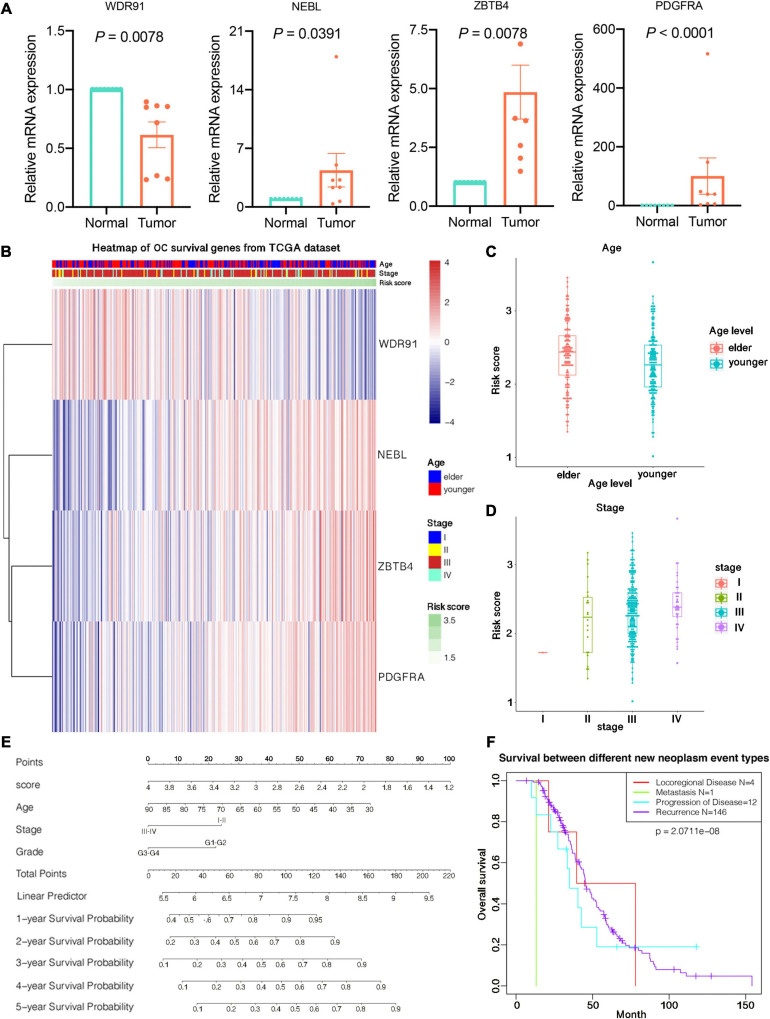
Relationship between prognostic risk score and clinicopathological characteristics of ovarian cancer. **(A)** Expression of 4 prognostic predictors in ovarian cancer tissues and normal tissues. **(B)** The expression profile of clinical characteristics of high and low risk groups. The horizontal axis is four candidate prognostic genes. Red and blue indicate the expression value of each gene corresponding to each sample, and the vertical axis is the sample. Each top is shown from top to bottom. Age, stage, and risk score information for each sample, where all samples are ranked according to risk score from low to high. **(C)** Differences in risk scores between patients of different ages. **(D)** Differences in risk scores between patients of different stages. **(E)** Predicting patients 1–5-years survival with risk scores using nomograms. **(F)** Survival curves between different new tumor event samples in TCGA datasets.

We examined the expression of 4 potential independent prognostic genes by qRT-PCR in 8 OC tissues and 8 normal ovarian tissues. We applied the unpaired *t* test to assess the differences between the two groups. The results showed that NEBL, PDGFRA and ZBTB4 were upregulated in OC tissues compared to in normal ovarian tissues, whereas WDR91 were downregulated in tumor tissues (Figure 3A). The mRNA expression results of qRT-PCR validation in 8 patients with OC were supporting effect on the establishment of the four-gene prognostic risk signature in OC.

### Prognostic Risk Score and Clinicopathological Features in OC

The heat map depicted the expression of four candidate m^6^A-related genes in high-risk patients (Figure 3B). We studied the clinicopathological characteristics of OC in the low- and high-risk groups, including age, stage, grade, new neoplasm event type, BRCA1 mutation status, BRCA2 mutation status, 19q13.2 CNV status, and 19q13.42 CNV status. The results illuminated that no meaningful difference was observed in the clinicopathological characteristics between the two groups containing stage, grade, new neoplasm event type, 19q13.2 CNV status, and mutation status of BRCA1 and BRCA2. However, there was significant difference found in 19q13.42 CNV status (*p* = 0.0390) and different ages (*p* = 0.0023).

Additionally, we also detected the relationship between risk score and each clinicopathological feature, and found that the risk score was significantly different among patients of different ages (*p* = 0.0043; Figure 3C), and the difference was also significant among patients at different stages (*p* = 0.0342; Figure 3D).

We used nomograms to further demonstrate the 1–5 years survival rate predicted by the risk score, including the risk of sample illness, age, stage, and other factors, visualizing the results of Cox regression analysis. A more accurate understanding of survival by looking at the total number of points corresponding to a sample of a certain condition, and the predicted survival rate of the sample in 1–5 years is shown in Figure 3E. Survival differences between different new tumor states are illuminated in Figure 3F, including one case of distant metastasis, four cases of local regional disease, 12 cases of disease progression, and 146 tumor recurrences.

### Consensus Clustering of m^6^A-Related Genes and Related Clinicopathological Characteristics and Survival Outcomes

**FIGURE 4:**
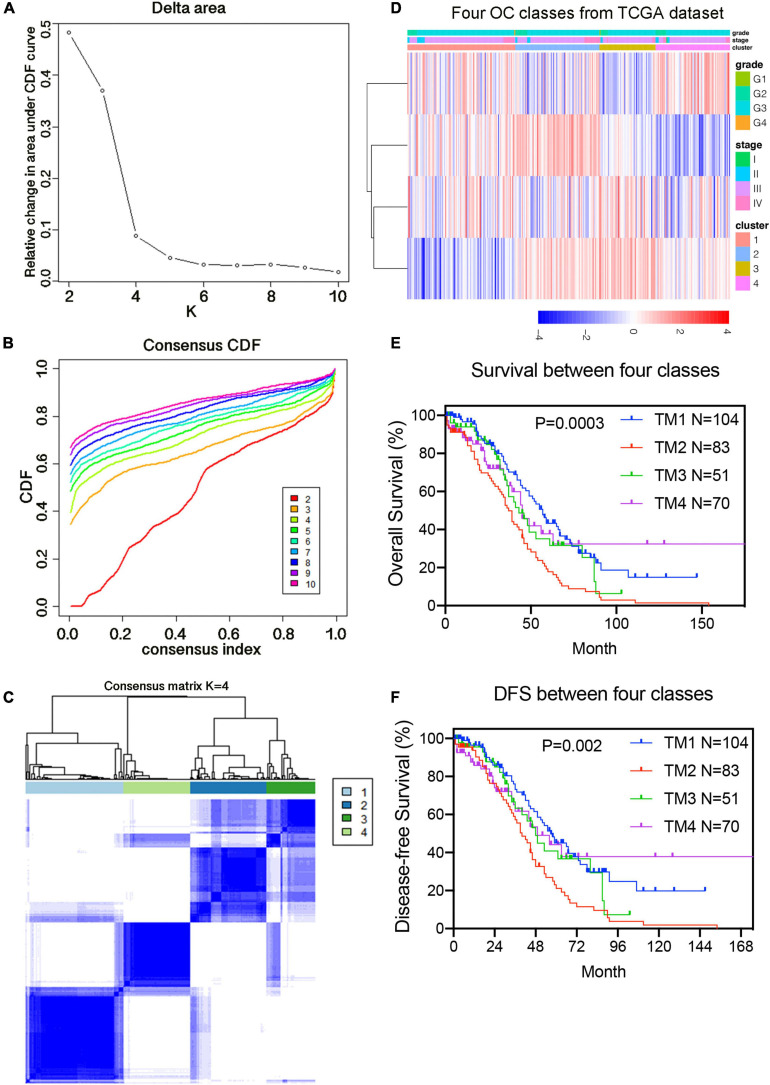
Identification of consensus clusters by 4 prognostic genes. **(A)** Relative change in area under CDF curve for *k* = 2–10 classified by the prognostic risk model. **(B)** Consensus clustering cumulative distribution function (CDF) for *k* = 2–10 classified by the prognostic risk model. **(C)** Consensus clustering matrix for k = 4. **(D)** Heat maps and significant clinical features of the four subgroups. **(E)** Kaplan–Meier overall survival (OS) curves for 308 samples in the four subgroups classified by 4 prognostic genes. **(F)** Kaplan–Meier disease-free survival (DFS) curves for 308 samples in the four subgroups classified by four prognostic genes.

To study the function of candidate m^6^A-related genes in OC, we separated the 308 TCGA OC samples into several subgroups using the expression similarity of 36 candidate m^6^A-related genes through the R “ConsensusClusterPlus” package. Based on the similarity of their expressions, k = 4 was the best k value for relatively stable clustering in a clustering range from 2 to 10 (Supplementary Figures 2A–C). All subgroups were named TM1, TM2, TM3, and TM4, respectively. After using Chi-Square test to analyze the clinicopathological characteristics of samples from the four subgroups of TM1 to TM4, it was found that the four subgroups had significant differences in age, stage and grade (*p* ≤ 0.05). But there was no notable difference in new neoplasm event type, BRCA1 mutation status, BRCA2 mutation status, 19q13.2 CNV status, and 19q13.42 CNV status. We further investigated the survival status between the four subgroups and uncovered that the difference in survival rates between these subgroups was not significant (*p* = 0.0998). What’s more, we separated the samples into several subgroups using the expression similarity of 4 prognostic genes. Utilizing consistent cluster analysis, the samples could be clearly divided into four categories with four prognostic genes, as shown in Figures 4A,B. Figure 4C demonstrated that the inflection point was larger when *k* = 4 and could be divided into four categories. What’s more, it was found that the new four subgroups had significant differences in age, stage and grade (*p* ≤ 0.05) (Figure 4D). We also investigated the survival status between the four subgroups and uncovered that the OS and DFS of TM2 group were significantly shorter than those of the other three groups (*p* = 0.0003, *p* = 0.002; Figures 4E,F).

### Interaction and Correlation Analysis of m^6^A Candidate Gene set

**FIGURE 5:**
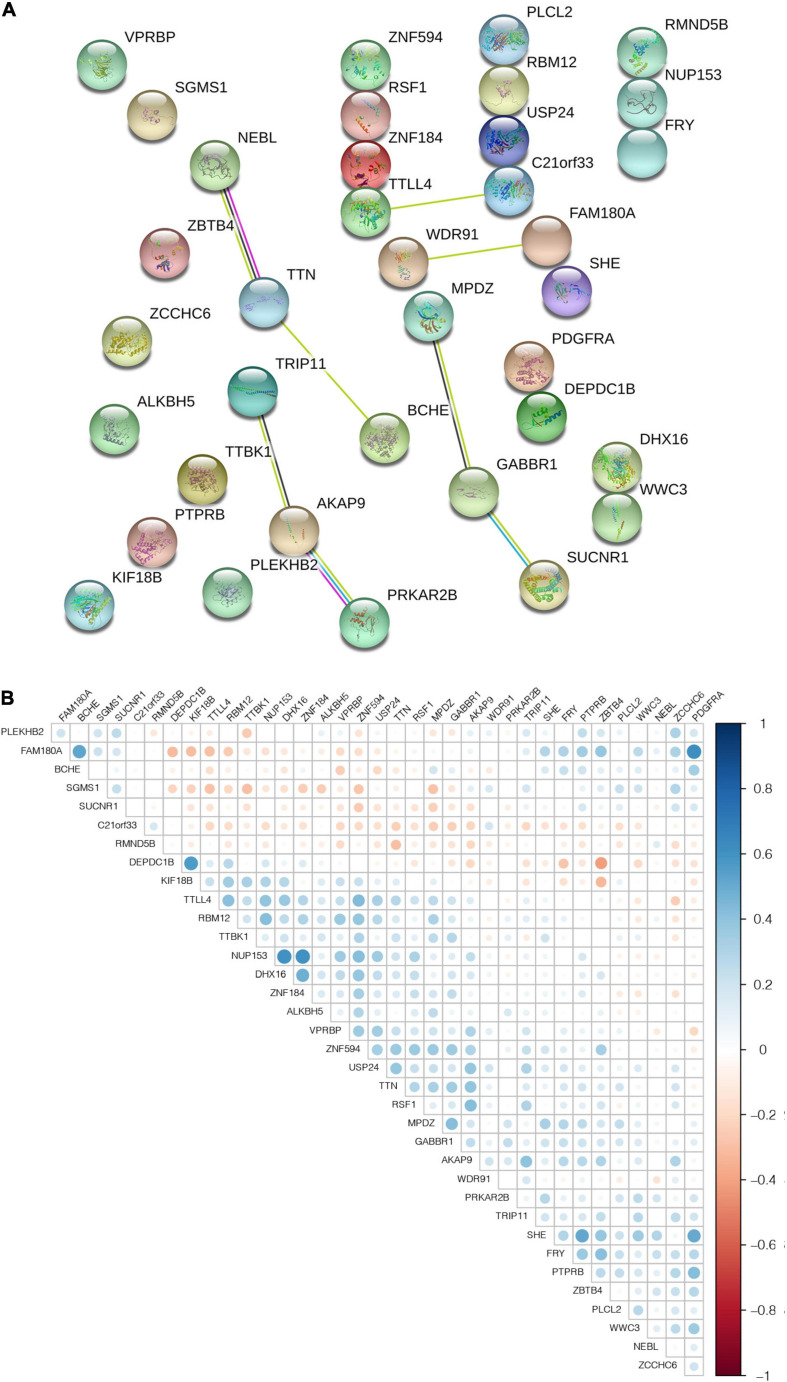
The interaction and correlation among m^6^A-related genes. **(A)** PPI network was constructed to evaluate the interaction among m^6^A-related genes. **(B)** The Pearson correlation analysis was used to determine the correlation among m^6^A-related genes.

To further understand the interactions between the 36 m^6^A-related genes, we analyzed the interactions and correlations between these genes. The interactions between the 36 m^6^A-related genes are demonstrated in Figure 5A. When the correlation coefficient threshold of the expression amount was set to | r | ≥ 0.3, and the *p*-value of the rank sum test was set to *p* ≤ 0.01, a total of 67 pairs of significantly correlated interaction factors were obtained (Supplementary Table 5). The results of the correlation analysis of all 36 candidate genes are shown in Figure 5B, in which the blue ones are positive and the red ones are negative.

### GO and KEGG Pathway Analyses of Differentially Expressed m^6^A-Related Genes

**FIGURE 6:**
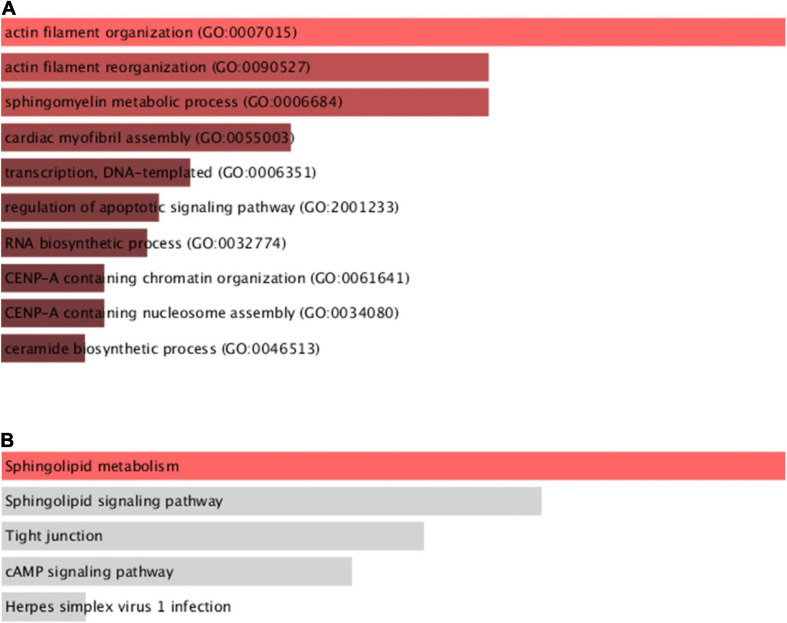
Functional enrichment analysis of differentially expressed genes between subgroups. **(A)** GO function analysis of differential genes among four subgroups. **(B)** KEGG pathway analysis of differential genes among four subgroups.

We performed differential gene analysis on candidate genes between the four subgroups of TM1 to TM4 to find m^6^A-related genes that were differentially expressed in the four subgroups. We identified 9 genes with significant differential expression among the 36 m^6^A-related genes using one-way ANOVA (*p* ≤ 0.05; Supplementary Table 6). We performed GO and KEGG functional annotation of differential genes using functional enrichment analysis tool (Supplementary Tables 7, 8). The results demonstrated that these differentially expressed genes were significantly related to biological functions including regulation of actin filament organization, sphingomyelin metabolism, transcription of DNA templates, and apoptotic signaling pathways (Figure 6A). Meanwhile, the differential genes were closely related to sphingolipid metabolism pathways, which were vital pathways involved in OC initiation (Figure 6B).

Among these differential genes, NEBL is a candidate gene with potential independent prognostic efficacy. The design of subsequent experiments for these genes can be considered to further investigate the potential pathological value of these genes and the design of new drug targets.

## Discussion

OC is one of the three most prevalent cancer types of female genitalia with the highest mortality rate, yet its pathogenesis is still unclear (Bamford and Webster, 2017). Thus, uncovering the intrinsic molecular mechanisms of OC tumorigenesis is of great significance. Aberrant expression of m^6^A-related genes has been identified in multiple cancers, which is proved to be closely related to pathological processes in cancer, including tumorigenesis, metastasis, and drug resistance (Ma et al., 2019). However, m^6^A-related genes have rarely been studied in OC. Thus, we conducted this study to investigate their roles in OC. Differential expression patterns for most m^6^A-related genes were identified in OC patients compared with controls based on RNA sequencing data from TCGA and ICGC. Significant difference was found in survival between the risk groups of samples. What’s more, a four-gene risk signature including NEBL, PDGFRA, WDR91 and ZBTB4 was confirmed, and this risk signature was considered as an independent predictor for prognosis of OC patients. Subsequently, we identified consensus clustering into four subgroups based on the expression of these four prognostic genes, and uncovered statistically significant differences of age, stage and grade among the subgroups. Moreover, the OS and DFS of TM2 group were shorter than those of the other three groups.

In terms of data from TCGA, m^6^A RNA methylation regulator ALKBH5 and other 35 m^6^A-related genes were dysregulated in OC patients, which indicated that m^6^A RNA methylation related genes might play a role in OC. Similar results were previously reported. Zhu et al. suggested that ALKBH5 regulated the proliferation, invasion and autophagy of OC cells via EGFR-PIK3CA-AKT-mTOR pathway and Bcl-2 (Zhu et al., 2019). In addition, METTL3 stimulates epithelial-mesenchymal transition (EMT) of cancer cells by stimulating receptor tyrosine kinase AXL, thereby enhancing the invasion and metastasis of OC (Hua et al., 2018). Han et al. (2020) studied the prognostic value of high-frequency genetic alterations of m^6^A RNA methylation regulators in OC. While we mainly focused on the prognostic value of m^6^A-related genes themselves for OC, and we identified a four-gene risk signature (Han et al., 2020). We also demonstrated distinct relationship between the expression of m^6^A-related genes and clinicopathological features in OC, such as 19q13.2_CNV, age and stage. 19q13.2_CNV was reported associated with decreased OC risk (Walker et al., 2017). What’s more, there were significant difference of age, stage and grade among the four subgroups using consensus clustering analysis, and the OS as well as DFS of TM2 group were shorter than those of the other three groups. The results above illustrate that m^6^A-related genes are likely to serve a vital function in the development/progression of OC, and further in-depth research is warranted to determine the underlying molecular mechanisms.

We then evaluated the effects of m^6^A-related gene alterations on survival of OC patients. Abundant studies have reported that the dysregulation of m^6^A-related genes was related to the prognosis of various cancers, such as breast cancer, gastric cancer, renal cell carcinoma, etc. (Liu et al., 2019; Su et al., 2019; Wang J. et al., 2020) In our study, we obtained 4 m^6^A-related genes that could be used as a feature of potential independent prognostic risk from the 36 candidate genes. Our four−gene prognostic signature implied that the expression level of NEBL, PDGFRA, ZBTB4 was negatively correlated with the survival of OC patients. Previous studies indicated that ZBTB4 and PDGFRA played roles in tumor progression, same as our prediction (Kim et al., 2012; Kang et al., 2015; Roussel-Gervais et al., 2017; Yu Y. et al., 2018; Ye et al., 2019). ZBTB4 is a mammalian DNA-binding protein acting as a transcriptional repressor (Yu Y. et al., 2018). ZBTB4 overexpression inhibits cancer cell proliferation and induces cell cycle arrest at G1 phase as well as apoptosis in Ewing’s sarcoma (Yu Y. et al., 2018). Roussel-Gervais et al. (2017) reported that decreased ZBTB4 expression correlated with the high genome instability among many frequent human cancers, which altered mitotic checkpoint, increased aneuploidy and promoted tumorigenesis. Similarly, underexpression of ZBTB4 is correlated with poor survival of breast cancer patients (Kim et al., 2012). PDGFRA is a gene encoding cell surface tyrosine kinase receptor (Ye et al., 2019). For PDGFRA, PDGFRA can promote downstream activation of the Notch1 pathway as well as the angiogenesis, proliferation and invasion of OC cells (Ye et al., 2019). And mutations in PDGFRA have been related to a variety of other cancers, including glioblastoma, melanoma, neuroendocrine carcinoma, etc. (Kang et al., 2015). However, the role of NEBL in cancer is complex. NEBL is a member of nebulin family of actin binding proteins (Zhang and Zhang, 2019). Zhang et.al found that NEBL promoted cancer cell proliferation, migration and invasion in cervical cancer by regulating PI3K/Akt pathway (Zhang and Zhang, 2019). On the contrary, upregulation of NEBL inhibited cancer cell migration and invasion and reversed TGF-β-induced EMT in prostate cancer (Wang et al., 2017). These indicate that m^6^A RNA methylation related genes might play different roles as tumor promoter or suppressor agents in different types of cancer, which need to be further elaborated.

Our GO analysis demonstrated that differentially expressed genes were significantly related to biological functions including regulation of actin filament organization and reorganization, sphingomyelin metabolic process, cardiac myofibril assembly, etc. Meanwhile, our KEGG analysis indicated that the differential genes were closely related to sphingolipid metabolism pathways. Previous studies have indicated that sphingolipid metabolic pathway participated in the regulation of vital cancer cellular processes, such as cell proliferation, migration, invasion, apoptosis and autophagy, playing an important role in the occurrence and development of OC (Hannun and Obeid, 2018; Jacob et al., 2018; Ogretmen, 2018). Meanwhile, key enzymes of sphingolipid metabolism were thought to be directly related to drug resistance in OC (Huang et al., 2016). These suggest that sphingolipid metabolism pathways may be a possible vital link in m^6^A-related genes involved in regulating OC.

However, there are also some potential limitations in the current study. First, there were no available datasets of tissues adjacent to OC from TCGA, and we used OC and normal ovarian tissue samples to verify the expression. Second, *p* ≤ 0.05 was considered statistically significant in our study which might have influence on the reliability and accuracy of the results (Colquhoun, 2017). Thus *p* ≤ 0.005 should be applied for our future research. Third, the m^6^A-related genes related to OC that we included were directly generated from the m^6^Avar database, and their regulatory functions might not have been verified enough. Subsequent experiments are needed to further verify the specific functioning mechanisms of these m^6^A-related genes, especially the 4 prognostic biomarkers we analyzed in this study.

## Conclusion

In conclusion, our study for the first time analyzed the expression of m^6^A-related genes in OC, and discovered that m^6^A-related genes were tightly relevant to the prognosis of OC patients, highlighting their roles as prognostic biomarkers in OC patients, as well as their potential functions in the occurrence and progression of OC. Further research is required to investigate the regulatory mechanisms of m^6^A modification in OC, which will help develop m^6^A RNA methylation related genes as valuable therapeutic targets.

## Data Availability Statement

Publicly available datasets were analyzed in this study, these can be found in The Cancer Genome Atlas (https://portal.gdc.cancer.gov/) the UCSC Xena Browser database (http://xena.ucsc.edu/).

## Ethics Statement

The studies involving human participants were reviewed and approved by the Health Research Ethics Board of the Shengjing Affiliated Hospital of China Medical University. Written informed consent for participation was not required for this study in accordance with the national legislation and the institutional requirements.

## Author Contributions

ZN and XW designed the study. ZN performed the data collection and the data analysis. ZN and LF drafted the manuscript. All authors read and approved the final version of the manuscript.

## Conflict of Interest

The authors declare that the research was conducted in the absence of any commercial or financial relationships that could be construed as a potential conflict of interest.

## Publisher’s Note

All claims expressed in this article are solely those of the authors and do not necessarily represent those of their affiliated organizations, or those of the publisher, the editors and the reviewers. Any product that may be evaluated in this article, or claim that may be made by its manufacturer, is not guaranteed or endorsed by the publisher.

## Footnotes

1
http://xena.ucsc.edu/

2
http://m6avar.renlab.org/

3
http://www.string-db.org/

4
http://amp.pharm.mssm.edu/Enrichr/

## Supplementary Material

Supplementary Figure 1 The expression levels of m^6^A methylation regulators of samples at different stages in TCGA ovarian cancer datasets (For stages: dark blue represents stage I, yellow represents stage II, red represents stage III, and light blue represents stage IV. For expression levels: red represents high expression, and blue represents low expression).

Supplementary Figure 2 Identification of consensus clusters by m^6^A-related genes. **(A)** Relative change in area under CDF curve for *k* = 2–10 classified by m^6^A-related genes. **(B)** Consensus clustering cumulative distribution function (CDF) for *k* = 2–10 classified by m^6^A-related genes. **(C)** Consensus clustering matrix for *k* = 4.
