# Supplementary figures and images for: Gene Signatures and Prognostic Values of N6-Methyladenosine Related Genes in Ovarian Cancer
Source: Front Genet. 2021 Aug 18;12:542457. doi: 10.3389/fgene.2021.542457 (PMC8416414; doi:10.3389/fgene.2021.542457)

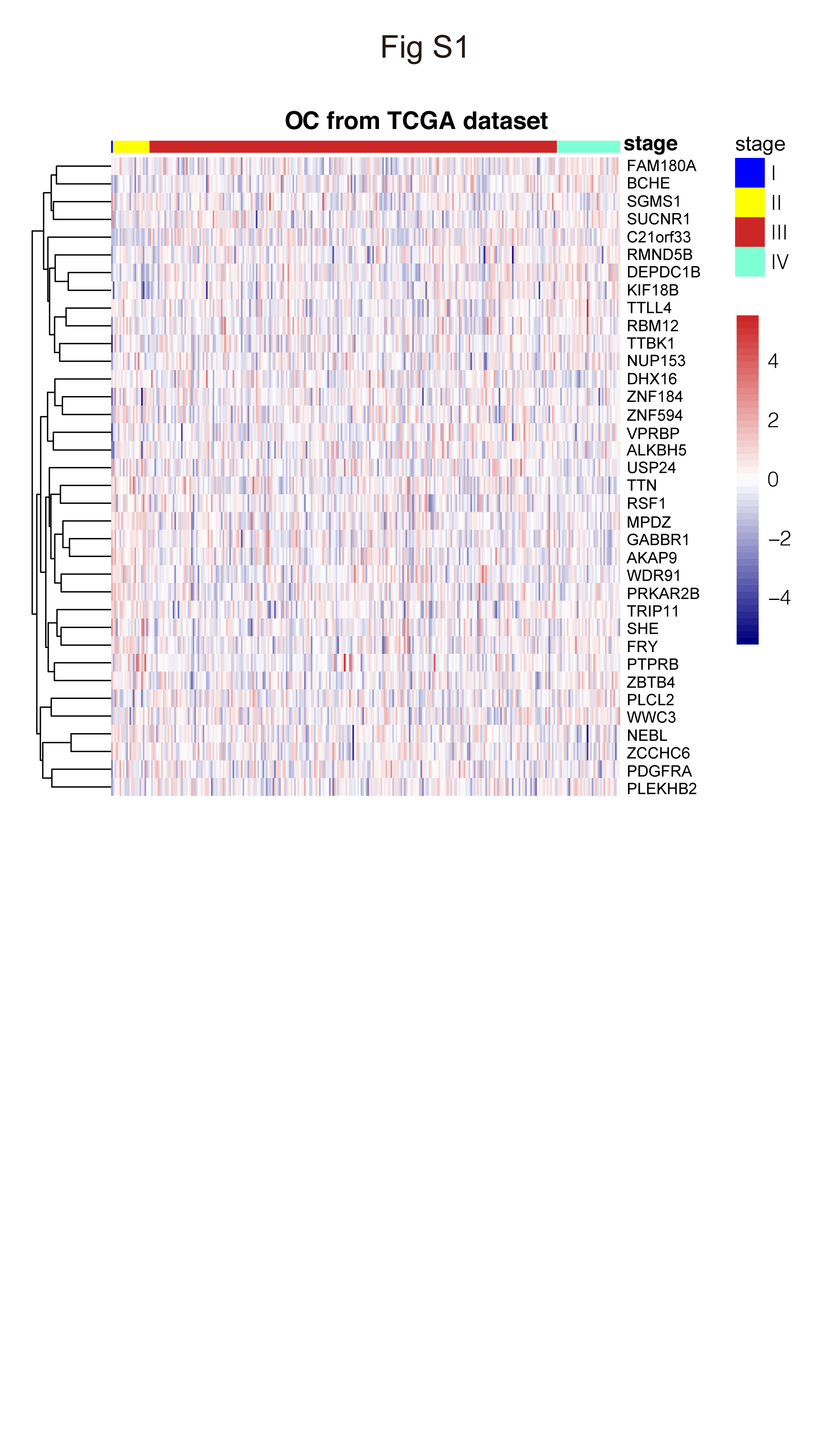

Supplement: Supplementary Figure 1 — The expression levels of m6A methylation regulators of samples at different stages in TCGA ovarian cancer datasets (For stages: dark blue represents stage I, yellow represents stage II, red represents stage III, and light blue represents stage IV. For expression levels: red represents high expression, and blue represents low expression). [file Image_1.TIF]

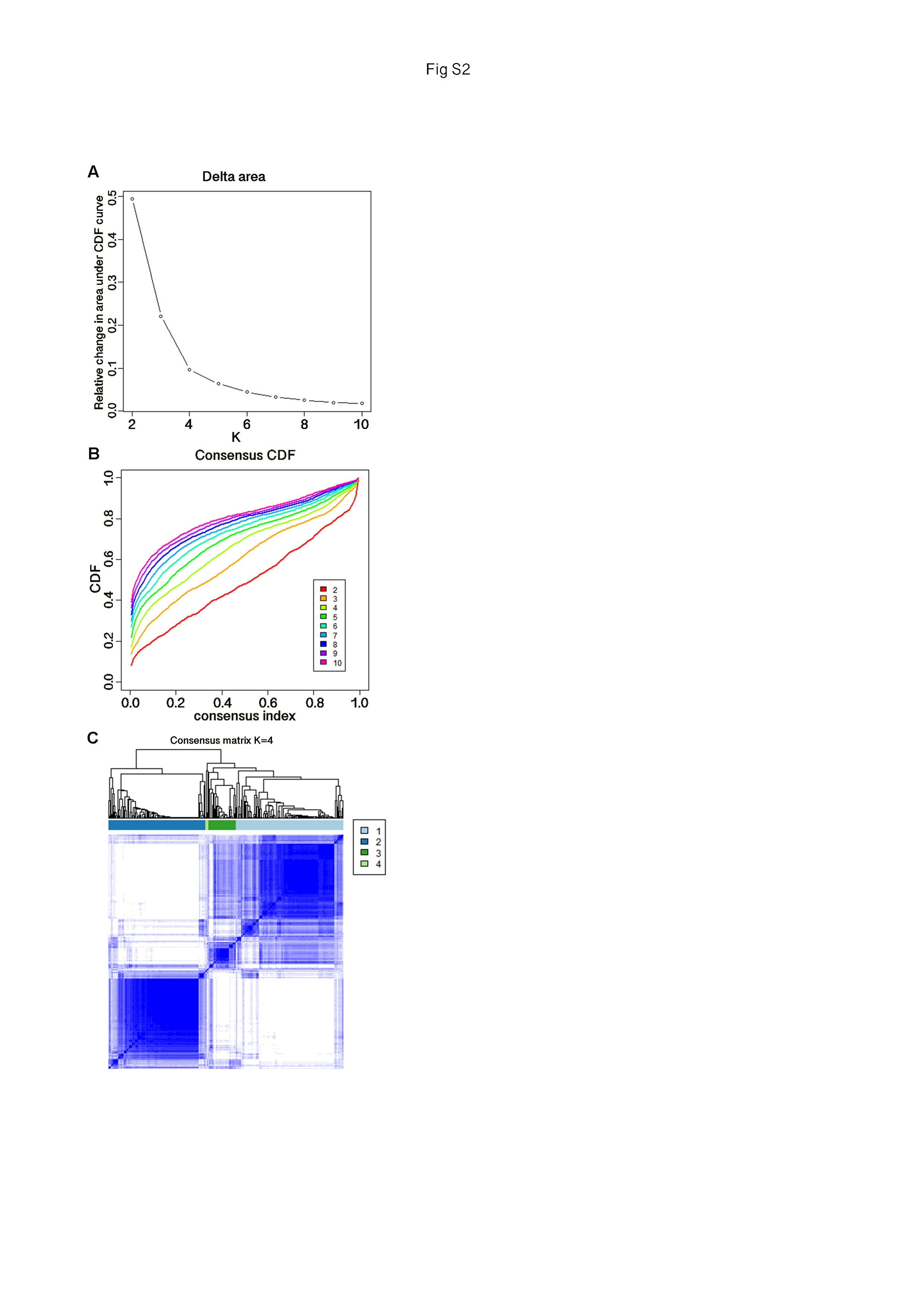

Supplement: Supplementary Figure 2 — Identification of consensus clusters by m6A-related genes. (A) Relative change in area under CDF curve for k = 2–10 classified by m6A-related genes. (B) Consensus clustering cumulative distribution function (CDF) for k = 2–10 classified by m6A-related genes. (C) Consensus clustering matrix for k = 4. [file Image_2.TIF]
